# Various Diseases and Clinical Heterogeneity Are Associated With “Hot Cross Bun”

**DOI:** 10.3389/fnagi.2020.592212

**Published:** 2020-11-20

**Authors:** Shuzhen Zhu, Hualing Li, Bin Deng, Jialing Zheng, Zifeng Huang, Zihan Chang, Yanjun Huang, Zhibo Wen, Yanran Liang, Mengjue Yu, Ling-Ling Chan, Eng-King Tan, Qing Wang

**Affiliations:** ^1^Department of Neurology, Zhujiang Hospital of Southern Medical University, Guangzhou, China; ^2^Department of Neurology, Shunde Hospital of Southern Medical University, Foshan, China; ^3^Department of Radiology, Zhujiang Hospital of Southern Medical University, Guangzhou, China; ^4^Department of Neurology, Sun Yat-sen Memorial Hospital of Sun Yat-sen University, Guangzhou, China; ^5^Department of Neurology, Chenghai People Hospital, Shantou, China; ^6^Department of Neurology, National Neuroscience Institute, Singapore General Hospital, Duke-NUS Medical School, Singapore, Singapore

**Keywords:** magnetic resonance imaging, disease spectrum, inflammation, MSA, stroke, ADEM, “hot cross bun” sign

## Abstract

**Objective:** To characterize the clinical phenotypes associated with the “hot cross bun” sign (HCBs) on MRI and identify correlations between neuroimaging and clinical characteristics.

**Methods:** Firstly, we screened a cohort of patients with HCBs from our radiologic information system (RIS) in our center. Secondly, we systematically reviewed published cases on HCBs and classified all these cases according to their etiologies. Finally, we characterized all HCBs cases in detail and classified the disease spectra and their clinical heterogeneity.

**Results**: Out of a total of 3,546 patients who were screened, we identified 40 patients with HCBs imaging sign in our cohort; systemic literature review identified 39 cases, which were associated with 14 diseases. In our cohort, inflammation [neuromyelitis optica spectrum disorders (NMOSD), multiple sclerosis (MS), and acute disseminated encephalomyelitis (ADEM)] and toxicants [toxic encephalopathy caused by phenytoin sodium (TEPS)] were some of the underlying etiologies. Published cases by systemic literature review were linked to metabolic abnormality, degeneration, neoplasm, infection, and stroke. We demonstrated that the clinical phenotype, neuroimaging characteristics, and HCBs response to therapy varied greatly depending on underlying etiologies.

**Conclusion**: This is the first to report HCBs spectra in inflammatory and toxication diseases. Our study and systemic literature review demonstrated that the underpinning disease spectrum may be broader than previously recognized.

## Introduction

Parkinson's disease (PD) is the second common age-related neurodegenerative disease with the motor and non-motor dysfunctions following Alzheimer's disease (AD) due to the progressive loss of dopaminergic neurons in the substantia nigra pars compacta (SNpc) (Qian and Huang, 2019; Xu et al., 2019; Beheshti et al., 2020; Yang et al., 2020). Conventional magnetic resonance imaging (cMRI) is often used to aid the diagnosis of some neurodegenerative diseases such as dementia, spinocerebellar ataxia (SCA), and multiple system atrophy (MSA), but its sensitivity is usually relative low (Massey et al., 2012; Goldman et al., 2014; Ozaki et al., 2015, 2019; Filippi et al., 2017; Agosta et al., 2018; Shi et al., 2018; Mazzucchi et al., 2019; Yang et al., 2019). In MSA and SCA patients, putaminal, and olivopontocerebellar abnormalities are present, usually with the appearance of a “hot cross bun” sign (HCBs) in the axial plane. The HCBs refers to a cruciform hyperintensity in the pons on T2-weighted magnetic resonance imaging (MRI). The signal is typically seen in MSA and SCA usually thought to result from the gliosis of pontocerebellar fibers (Gulati et al., 2007). However, recent evidence suggests that HCBs is not a specific sign for MSA and SCA, but it can be observed in other diseases along with various clinical phenotypes (Muqit et al., 2001; Soares-Fernandes et al., 2009; Yadav et al., 2011; Jain et al., 2013, 2014; Padmanabhan et al., 2013; Pedroso et al., 2013; Roh et al., 2013; Zhang et al., 2013; Pan et al., 2015; Ishikawa et al., 2016; Wang et al., 2016). To our knowledge, few studies have systematically depicted the disease spectrums with HCBs, analyzed the clinical heterogeneity, and evaluated the disease progression and clinical outcomes (van Eimeren et al., 2019).

To address the gaps in knowledge, we firstly conducted the analysis regarding a detailed characterization on HCBs by screening patients in our center and then performed a systematic literature review on published cases with imaging data. Finally, we described the heterogeneity of clinical phenotypes and HCBs neuroimaging features in various HCB-positive diseases.

## Methods

### Study Design, Participants, and Clinical Assessment

**Figure 1:**
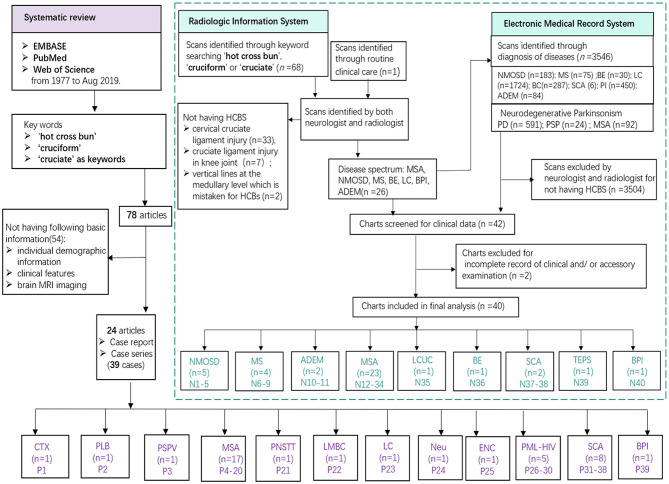
Study flowchart. Various diseases founded in previously published literatures (purple) and our cohort (green). MSA, multiple system atrophy; NMOSD, neuromyelitis optica spectrum disorders; MS, multiple sclerosis; ADEM, acute disseminated encephalomyelitis; SCA, spinocerebellar ataxia; DLB, dementia with Lewy body; LCUC, lung cancer with undefined cause; BE, brain stem encephalitis; TEPS, toxic encephalopathy caused by phenytoin sodium; BPI, bilateral pons infarction; CTX, cerebrotendinous xanthomatosis; PSPV, parkinsonism secondary to presumed vasculitis; PNSTT, paraneoplastic neurological syndrome due to burned-out testicular tumor; LMBC; leptomeningeal metastases of breast cancer; LC, lung cancer; Neu, neurosarcoidosis; ENC, encephalitis; PML-HIV, HIV-related progressive multifocal leukoencephalopathy.

Newly screened patients in our cohort were collected through the radiologic information system (RIS) and electronic medical records system (EMRS) by searching the terms “hot cross bun,” “cruciform,” or “cruciate” in our center. Out of 68 scans identified through simple keyword searching on the RIS, 42 were excluded for not having HCBs. Among the 42 cases, 33 had cervical cruciate ligament injury, 7 had cruciate ligament injury in knee joint, and 2 had vertical lines at the medullary level which is not typical for HCBs. We found a new patient diagnosed with ADEM with HCBs in the routine clinical care; however, this patient was lately confirmed by a radio-neurologist. The detailed methods of newly screened patient inclusion are summarized in Figure 1. Formal consents for publication were obtained from newly screened patients whose detailed clinical data were reported. This clinical study in our center was approved by the ethics committees of the Zhujiang Hospital of Southern Medical University, China (2018-SJNK-002). The data were collected from published cases and newly screened cases in the hospital over a 6-year period (January 2013 to May 2019).

### Image Analysis

Brain MRI examination in our center was performed using a 3-T MR scanner (Achieva, Siemens Healthineers, Germany) with a 16-channel head coil. T1, T2, and T2 fluid-attenuated inversion recovery (FLAIR) sequences were used to identify HCBs. The acquisition parameters for each sequence were indicated as follows: T1-weighted imaging (coil selection: SENSE-NV-16; FOV: 22 ×19, TR 2,000 ms, TE 20 ms); T2 weighted spin echo sequence (coil selection: SENSE-NV-16; FOV: AP 215 mm, RL 197 mm, FH 125 mm; voxel size AP 0.575 mm, RL 0.72 mm, slice thickness 6 mm, stack fold-over direction RL, TR 3,000 ms, TE 80 ms); T2-Flair imaging (coil selection: SENSE-NV-16; FOV: 22 ×19, TR 11,000 ms, TE 125 ms). The progression of the HCBs was assessed according to criteria reported by Horimoto et al. (2002). The HCBs was graded as follows: no change appeared = 0; a vertical T2 high-intensity line began to appear = 1; only a clear vertical line appeared = 2; a horizontal line began to appear following a vertical line appearance = 3; both a clear horizontal and a vertical line appeared (cross line completed) = 4; and the ventral pons in front of the horizontal line showed T2 high intensity or the ventral pons decreased in size with pons base atrophy = 5.

### Clinical Assessment

For our clinical cohort, demographic characteristics, clinical variables, and neuroimaging features of all newly screened patients were assessed: age, gender, duration of disease, gait instability, movement retardation, static tremor, rigidity, dysuria, cognitive decline, cerebellar language, nystagmus, ataxia of limbs, difficulty in straight-line walking, and neuroimaging signs.

### Search Strategy, Selection Criteria, and Standard Patient Collection

We conducted a systematic review using EMBASE, PubMed, and Web of Science from 1977 to Aug 2019. Published cases were sought using “hot cross bun” or “cruciform” or “cruciate” as keywords, and 78 articles (Supplementary References) were found and 24 relative articles (case reports/series in which each case included the following information: individual demographic information, clinical features and brain MRI) were analyzed (Muqit et al., 2001; Soares-Fernandes et al., 2009; Yadav et al., 2011; Brooks, 2012; Jain et al., 2013, 2014; Padmanabhan et al., 2013; Pedroso et al., 2013; Roh et al., 2013; Goldman et al., 2014; Deguchi et al., 2015; Ozaki et al., 2015, 2019; Pan et al., 2015; Ishikawa et al., 2016; Wang et al., 2016; Nagpal and Agarwal, 2017; Way et al., 2019; Carre et al., 2020), and 39 reporting cases were reviewed. For published cases resulting from the systematic review, we collected data on disease diagnosis, age, gender, disease duration, symptoms (parkinsonism or cerebellar ataxia or urination dysfunction), imaging features, treatment, and the response of the HCBs to treatment. Detailed data are shown in Table 1 and Supplementary Table 1.

**Table 1:** Demographic characteristics, clinical manifestation, and imaging features of HCBs patients.

	**Metabolism (*n* = 1)**	**Inflammation (*n* = 11)**	**Degeneration (*n* = 42)**	**Neoplasm (*n* = 5)**	**Infection (*n* = 7)**	**Hereditary (*n* = 10)**	**Toxication (*n* = 1)**	**Stroke (*n* = 2)**	**χ^2^**	**P^**a**^**
**Sex**										
Women	0	6/11 (54.5%)	33/42 (78.6%)	3/5 (60.0%)	3/7 (42.9%)	2/10 (20.0%)	1	1	2.59	0.134^‡^
Men	1	5/11 (45.5%)	9/42 (21.4%)	2/5 (40.0%)	4/7 (57.1%)	8/10 (80.0%)	0	1		
**Age (y)**	25	49 (40–55)	60 (52–64)	48 (40–68)	30 (13–37)	48 (37–71)	24	66 (61–66)		0.004^§^
**Duration of disease (m)**	510	1 (0.47–5)	36 (12–48)	13.8 (3.6–13.8)	2.0 (2.0–6.8)	66 (21–180)	240	8 (4–8)		<0.001^§^
**Clinical manifestation (n)**										
Limb weakness	0/1 (0.0%)	7/11 (63.6%)	0/41 (0.0%)	0/5 (0.0%)	0/6 (0.0%)	0/10 (0.0%)	1/1 (100%)	1/2 (50.0%)	30.15	<0.001^‡^
Limb numbness	0/1 (0.0%)	1/11 (9.1%)	0/41 (0.0%)	0/5 (0.0%)	0/6 (0.0%)	1/10 (10.0%)	0/1 (0.0%)	1/2 (50.0%)	3.8	0.212^‡^
Vision blurred	0/1 (0.0%)	4/11 (36.4%)	0/41 (0.0%)	0/5 (0.0%)	0/6 (0.0%)	0/10 (0.0%)	0/1 (0.0%)	0/2 (0.0%)	16.15	0.002^‡^
Parkinsonism	0/1 (0.0%)	0/11 (0.0%)	21/41 (51.2%)	0/5 (0.0%)	0/6 (0.0%)	3/10 (30.0%)	0/1 (0.0%)	0/2 (0.0%)	9.45	0.002^‡^
Cerebellar ataxia	1/1 (0.0%)	1/11 (9.1%)	30/41 (73.1%)	2/5 (40.0%)	4/6 (66.7%)	10/10 (100%)	1/1 (100%)	0/2 (0.0%)	14.79	<0.001^‡^
**MRI feature**										
T1	0/1 (0.0%)	4/11 (36.4%)	1/23 (4.3%)	0/1 (0.0%)	2/4 (50.0%)	2/2 (100%)	0/1 (0.0%)	0/2 (0.0%)	6.08	0.029^‡^
T2	1/1 (100.0%)	11/11 (100%)	36/36 (100%)	5/5 (100%)	6/6 (100%)	10/10 (100%)	1/1 (100.0%)	2/2 (100.0%)	/	/
T2 Flair	0/1 (0.0%)	3/11 (27.3%)	4/36 (11.1%)	0/1 (0.0%)	4/6 (66.7%)	2/3 (66.7%)	0/1 (0.0%)	0/2 (0.0%)	3.42	0.341^‡^
Grade										
1	0/1 (0.0%)	5/11 (45.5%)	7/36 (19.4%)	0/5 (0.0%)	0/6 (0.0%)	1/10 (10.0%)	0/1 (0.0%)	0/2 (0.0%)	3	0.118^‡^
2	0/1 (0.0%)	0/11 (0.0%)	5/36 (13.9%)	0/5 (0.0%)	0/6 (0.0%)	3/10 (30.0%)	0/1 (0.0%)	0/2 (0.0%)	1.71	0.322^‡^
3	1/1 (0.0%)	4/11 (36.4%)	13/36 (36.1%)	2/5 (40%)	4/6 (66.7%)	6/10 (60.0%)	1/1 (0.0%)	2/2 (100%)	0	1.000^‡^
4	0/1 (0.0%)	2/11 (18.2%)	8/36 (22.2%)	3/5 (60%)	2/6 (33.3%)	0/10 (0.0%)	0/1 (0.0%)	0/2 (0.0%)	0.08	1.000^‡^
5	0/1 (0.0%)	0/11 (0.0%)	3/36 (8.3%)	0/5 (0.0%)	0/6 (0.0%)	0/10 (0.0%)	0/1 (0.0%)	0/2 (0.0%)	0.98	1.000^‡^
MCP-H	0/1 (0.0%)	2/11 (18.2%)	4/36 (11.1%)	3/5 (60%)	4/6 (66.7%)	5/10 (50.0%)	0/1 (0.0%)	0/2 (0.0%)	0.38	0.614^‡^
P-A	1/1 (100.0%)	0/11 (0.0%)	31/36 (86.1%)	4/5 (80%)	1/7 (14.3%)	8/10 (80.0%)	0/1 (0.0%)	0/2 (0.0%)	27.83	<0.001^‡^
MCP-A	0/1 (0.0%)	0/11 (0.0%)	35/36 (97.2%)	4/5 (80%)	1/7 (14.3%)	9/10 (90.0%)	1/1 (100.0%)	0/2 (0.0%)	41.89	<0.001^‡^
Cerebellar atrophy	0/1 (0.0%)	0/11 (0.0%)	36/36 (100%)	4/5 (80%)	2/7 (28.6%)	9/10 (90.0%)	1/1 (100.0%)	0/2 (0.0%)	47	<0.001^‡^
**Treatment& response**										
Etiological Treatment	Chenodeoxycholic acid	Methylprednisolone	N/A	N/A	Immunosuppressive	N/A	N/A	Antiplatelet		
Response	N/A	3/3 (100.0%)	N/A	N/A	1/1 (100.0%)	N/A	N/A	N/A		

a *Comparison was done between inflammation and degeneration groups. Data are n (%), median (IQR)*.

*†Based on χ2 test with significance level of 0.05 (two-tailed). Based on Fisher's exact test (if any cell has fewer than 10 observations) with level of significance set to 0·05 (two-tailed)*.

§ *Based on Mann–Whitney test with significance level of 0.05*.

*MCP-H, middle cerebellar peduncle hyperintensity; IQR, interquartile range; P-A, pons atrophy; MCP-A, middle cerebellar peduncle atrophy; m, month; y, year*.

### Data Availability

Anonymized data will be shared by reasonable request from any qualified investigator.

## Results

### Disease Spectra of the HCBs

We identified 79 patients including 40 newly identified cases from 3,546 participants and systematically reviewed 39 published reports (from 24 articles). As **Figure 3** shows, the underlying disease spectrum was varied including metabolism abnormality (cerebrotendinous xanthomatosis, Jain et al., 2013), degeneration multiple system atrophy, probable dementia with Lewy bodies (Way et al., 2019) and parkinsonism secondary to presumed vasculitis (Muqit et al., 2001), neoplasm (neurosarcoidosis) (Nagpal and Agarwal, 2017), paraneoplastic neurological syndrome due to burned-out testicular tumor (Ishikawa et al., 2016), leptomeningeal metastases of breast cancer (Pan et al., 2015), leptomeningeal carcinomatosis (Zhang et al., 2013), **i**nfection [encephalitis (Gan et al., 2018), progressive multifocal leukoencephalopathy with HIV seropositivity (Padmanabhan et al., 2013; Jain et al., 2014), variant Creutzfeldt–Jakob disease (Soares-Fernandes et al., 2009)], **h**ereditary [hereditary cerebellar ataxia including SCA (Pedroso et al., 2013; Wang et al., 2016)], and **s**troke [bilateral pons infarction (Roh et al., 2013)]. Among these, MSA and SCA were the most common etiologies. To our knowledge, we highlight for the first time **i**nflammation (NMOSD, MS, and ADEM) and toxic damage [toxic encephalopathy caused by phenytoin sodium (TEPS)] as new disease association with HCBs (Figures 1, **3** and Table 1). In addition, a patient with lung cancer had HCBs in the pons, but the etiologic cause is not clear.

### Demographic Characteristics and Clinical Phenotypes in HCBs-Positive Patients

Demographic and clinical characteristics for each case are listed in Table 1 (Muqit et al., 2001; Srivastava et al., 2005; Takao et al., 2007; Massano et al., 2008; Suresh Chandran et al., 2008; Soares-Fernandes et al., 2009; Yadav et al., 2011; Recio Bermejo et al., 2012; Gooneratne et al., 2013; Jain et al., 2013, 2014; Padmanabhan et al., 2013; Pedroso et al., 2013; Roh et al., 2013; Zhang et al., 2013; Namekawa et al., 2015; Pan et al., 2015; Das et al., 2016; Ishikawa et al., 2016; Lin et al., 2016; Wang et al., 2016; Nagpal and Agarwal, 2017; Gan et al., 2018; Way et al., 2019); the published and newly screened MSA patients with HCBs in our center are listed in Supplementary Table 1.

#### Demographic Characteristics

The patients in our clinical cohort included 12 males and 28 females with a median age of 55 years (range of 21 to 79 years, interquartile range, 14.5 years), median age of onset of 52 years (range of 20 to 77 years, interquartile range, 11.5 years), and median duration of disease of 0.7083 years (range of 0.01 to 5 years, interquartile range, 2.79 years). Systematic review showed that published cases (Supplementary Table 1) were reported mostly in Asian countries; 21 out of 39 patients were reported from India, 5 from Japan, 4 from China (mainland), 1 from South Korea, 1 from China (Taiwan), 3 from Spain, 2 from Portugal, 1 from the UK, and 1 from Brazil. These included 15 males and 24 females with a median age of 59 years (range of 3 to 85 years, interquartile range, 31 years) and a median duration of disease of 3 years (range of 0.17 to 36 years, interquartile range, 4.25 years) (Supplementary Table 1).

#### Clinical Phenotypes

**Figure 2:**
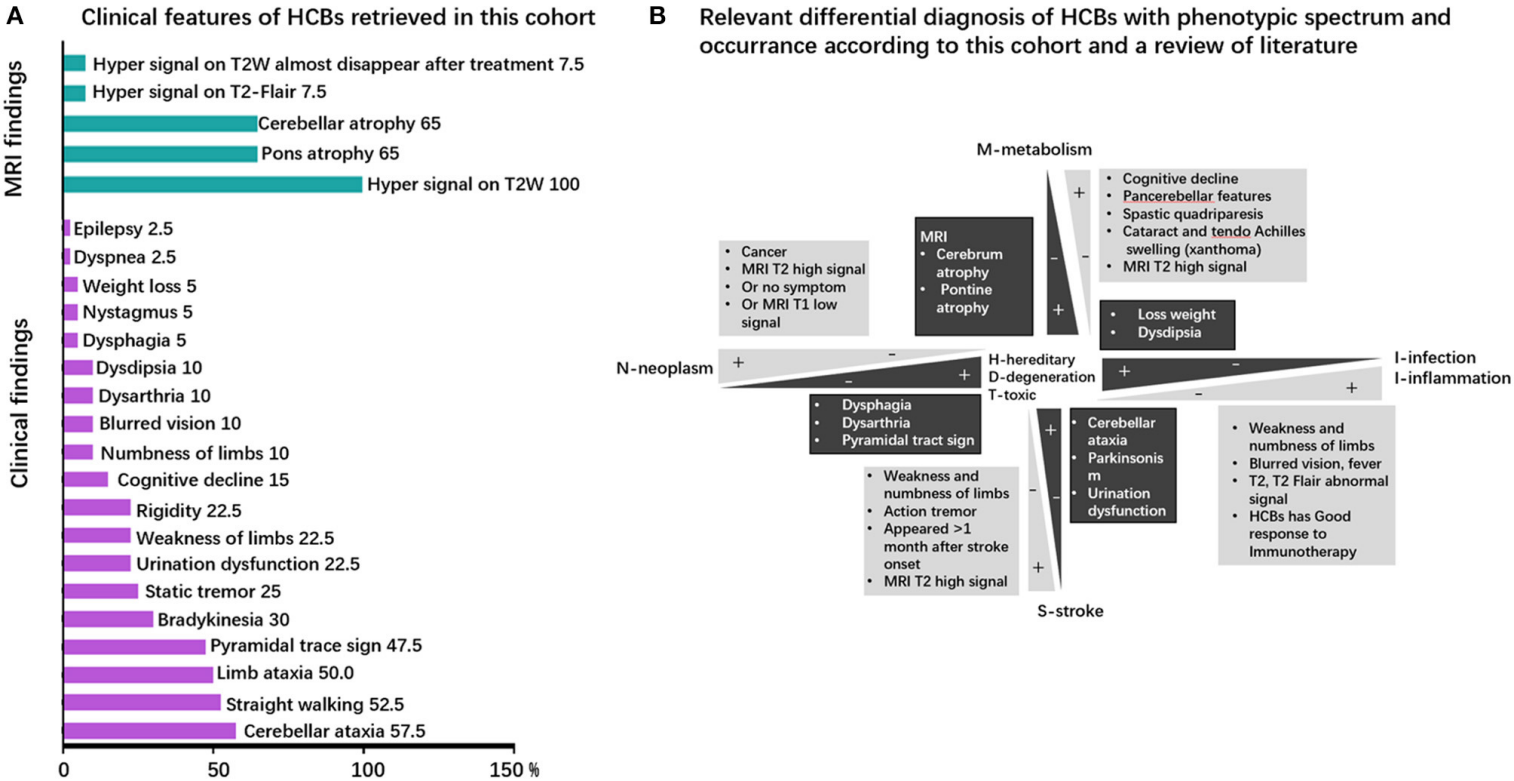
Clinical phenotype and neuroimaging spectrum and the neuroimaging–clinical phenotype correlations of HCBs. **(A)** Clinical phenotypic spectrum of the HCBs. The clinical and imaging findings of the HCBs-positive diseases with prevalence rates (in percent) of specific features. MRI findings are colored in green, and clinical signs and symptoms are highlighted in purple. **(B)** The relevant differential diagnosis of HCBs. The clinical phenotype of the HCBs overlapped among different diseases, and some diseases shared some common features. The main differential diagnoses of the HCBs-related diseases are depicted. Discriminating features are highlighted.

Clinical symptoms and signs of published cases (Supplementary Table 1) included cerebellar ataxia (unstable gait), parkinsonism, urinary urgency or incontinence, dementia, dysarthria, and action tremor. The clinical phenotype in our cohort comprised of unstable gait (57.5% of patients), cerebellar ataxia (52.5%), limb ataxia (50%), pyramidal tract sign (47.5%), bradykinesia (30%), cerebellum language (25%), static tremor (25%), rigidity (22.5%), urination dysfunction (22.5%), weakness of limbs (22.5%), cognitive decline (15%), numbness of limb (10%), dysdipsia (10%), dysarthria (10%), blurred vision (10%), dysphagia (5%), nystagmus (5%), loss weight (5%), epilepsy (2.5%), and dyspnea (2.5%). Cerebellar ataxia, the most common symptom associated with pontine nucleus and cerebellopontine tract injury, is usually observed in neurodegenerative, hereditary, and infectious diseases. It can also be found in metabolic diseases. However, despite the clear presence of the HCBs, cerebellar ataxia is not often observed in some diseases, such as neoplasm-related diseases, inflammatory diseases, and stroke. The clinical phenotypes are presented in Figure 2A and Supplementary Table 1 and the relevant differential diagnosis of HCBs are presented in Figure 2B.

### Neuroimaging Findings in HCBs-Positive Patients

We examined the MRI images from 40 newly cases from our cohort and reviewed 39 available published cases. The diagram of neuroimaging features, such as anteroposterior diameter of the pons, thickness of the middle peduncle of cerebellum, high signal in the middle cerebellar peduncle, and atrophy of cerebellum, is shown in Figure 4. The representative images of HCBs are shown in **Figure 5**. A single patient for each pathology is represented. The display of HCBs in each sequence is shown in **Figure 5** and Supplementary Table 1. As **Figure 5A** shows, the signal of the HCBs changed along with the occurrence and progression of disease in a patient diagnosed with MSA, a disease belonging to neurodegenerative disorder. At two years after symptom onset, HCBs (grade 3) was shown in the pons (**Figure 5A**a), which upgraded to level 4 (**Figure 5A**b) and 5 (**Figure 5A**c). The grade of HCBs gradually increased as the disease progresses. In addition to MSA, HCBs is also commonly found in hereditary disease, such as SCA (**Figure 5B**). For SCA patients, HCBS can be displayed in T1, T2, and T2 flair images as shown in Table 1. In this study, we firstly found that HCBs can also appear in inflammatory diseases (**Figure 5C**). In a case of NMOSD, after 9 years of relapse, we found HCB sign in the pons, with a low signal on T1 and a high signal on T2 and T2 flair (**Figure 5C**a). After effective immunosuppressive therapy, the signal of HCBs gradually weakened (**Figure 5C**b) until it almost disappeared (**Figure 5C**c). However, after the recurrence of the disease, HCBS appeared again in the pons (**Figure 5C**d). HCBs was also found in patients with other inflammatory diseases, such as MS (**Figure 5C**e) and ADEM (**Figure 5C**f). In this study, we also found that HCBs appeared in cases with neoplasm diseases **Figure 5D**. As **Figure 5D**a shows, clear HCBs was found on the T2 and T2 flair sequence in a case with lung cancer. HCBs can also emerge in cases with stroke. As **Figure 5E** shows, in a case with bilateral middle cerebral peduncle infarction, both T1 and T2 images showed HCBs. Infection is another type of disease with HCBs (**Figure 5F**). We reported a case with encephalitis, who had no abnormal signal in the pons (**Figure 5F**a) 2 months after the onset of disease and then had sporadic lesions 3 months later (**Figure 5F**b) and finally showed clear HCBs in the pons 6 months after the onset of encephalitis (**Figure 5F**c). Toxicity is one of the pathologic mechanisms that led to HCBs formation. We reported a girl with toxic encephalopathy caused by phenytoin sodium, whose pons showed a weak HCB signal on T2 and T2 flair images (**Figure 5G**).

#### The Signal Characteristics of the HCBs

The signal characteristics of the HCBs in MRI are not always the same in various diseases. In our study, MRI images were displayed in 72 cases. HCBs was found in all 72 cases on the T2 image sequence. In addition, HCBs was observed in 9 cases on T1-w images, and 13 cases on the T2 flair sequence. Among the 9 cases, 4 cases were from the inflammation group, 2 from the infection group, 2 from the hereditary group, and 1 from the degenerative disease group. In the inflammation group, 4 out of 11 patients had an HCB signal on the T1 image; the positive rate reached 36.4%, which was much higher than those in the degenerative disease group (36.4% vs. 4.3%, p=0.029).

#### Accompanying Imaging Features of the HCBs

The HCBs was accompanied by additional relevant neuroimaging findings, such as high signal in the middle cerebellar peduncle (MCP-H) and atrophy of the pons and cerebellum. The detailed data is shown in Table 1 and Supplementary Table 1. 18 out of 72 cases had MCP-H, which was more commonly seen in patients with neoplastic diseases than degenerative diseases (60 vs. 11%, *p* <0.001). Pons atrophy, MCP atrophy, and cerebellar atrophy were mostly present in patients with neurodegenerative, neoplasm, and hereditary diseases; however, they less seen in patients with inflammation and infection diseases. Symmetric ribbon-shaped abnormal signals (T1 hypointense, T2 and T2 FLAIR hyperintense) near the lenticular nucleus were found in a patient with toxic encephalopathy caused by phenytoin sodium (TEPS). However, the imaging of this patient showed significant cerebellum atrophy but almost normal pons volume, which differed largely from the characteristics of MSA-C (**Figure 5G**b).

#### HCBs in Response to Etiological Treatment

In neurodegenerative diseases, the signal of the HCBs gradually became evident with the progression of the diseases. However, in some diseases, such as NMOSD, the HCBs almost disappears after immunotherapy.

Taken together, the clinical and imaging findings related to the HCBs varied greatly depending on underlying diseases (**Figure 5**).

## Discussion

To our knowledge, this is the first detailed systematic review of the imaging and clinical features of a large cohort (comprising of equal proportion of newly identified and published cases) of patients with MRI evidence of HCBs. We highlight the following novel observations: (1) The clinical spectrum of diseases associated with HCBs is wider than previously thought. (2) The clinical phenotype, neuroimaging characteristics, and resolution of HCBs were varied depending on the underpinning diseases. (3) We identified novel disease associations including NMOSD, MS, and ADEM and toxin-related neurological damage.

**Figure 3:**
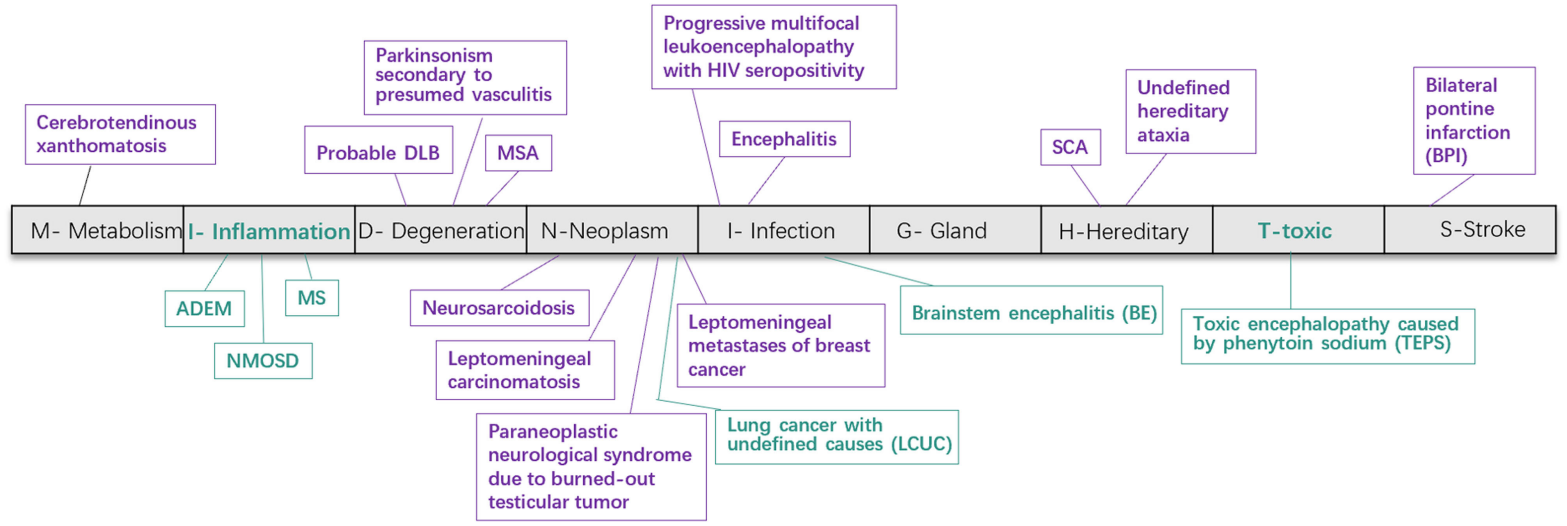
Disease spectrum of the HCBs. The HCBs-positive diseases were classified into nine categories according to the “MIDNIGHTS” diagnostic principle. This figure shows various diseases found in previously published literature (purple) and newly screened in our cohort (green). HCBS, “hot cross bun” sign; RIS, radiologic information system; MSA, multiple system atrophy; NMOSD, neuromyelitis optica spectrum disorders; MS, multiple sclerosis; ADEM, acute disseminated encephalomyelitis; BE, brainstem encephalitis; LCUC, lung cancer with undefined cause; BPI, bilateral middle cerebral peduncle infarction; SCA, spinocerebellar ataxia; TEPS, toxic encephalopathy caused by phenytoin sodium.

Pathological studies have confirmed that the HCBs in MSA is caused by gliosis of pontocerebellar fibers (Gulati et al., 2007). However, our study showed that HCBs is not the unique sign of MSA and can appear in a variety of diseases (Massey et al., 2012; Louapre and Lubetzki, 2015; Ozaki et al., 2015, 2019; Louapre et al., 2017; Filippi et al., 2019; van Eimeren et al., 2019). These included metabolic dysfunction, degeneration, neoplasm, infection, stroke, inflammation, and toxin-linked encephalopathy. Of interest is the involvement of inflammatory demyelination and toxic factors that can selectively damage the cerebellopontine tract, leading to HCBs emergence (Figures 3, 5).

**Figure 4:**
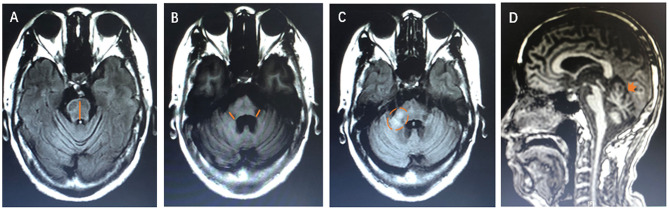
The diagram of neuroimaging features. **(A)** The orange line shows the measurement of anteroposterior diameter of the pons. **(B)** The orange line shows the thickness of the middle peduncle of cerebellum. **(C)** The orange dotted circle shows the high signal in the middle cerebellar peduncle. **(D)** The orange arrow shows atrophy of cerebellum.

**Figure 5:**
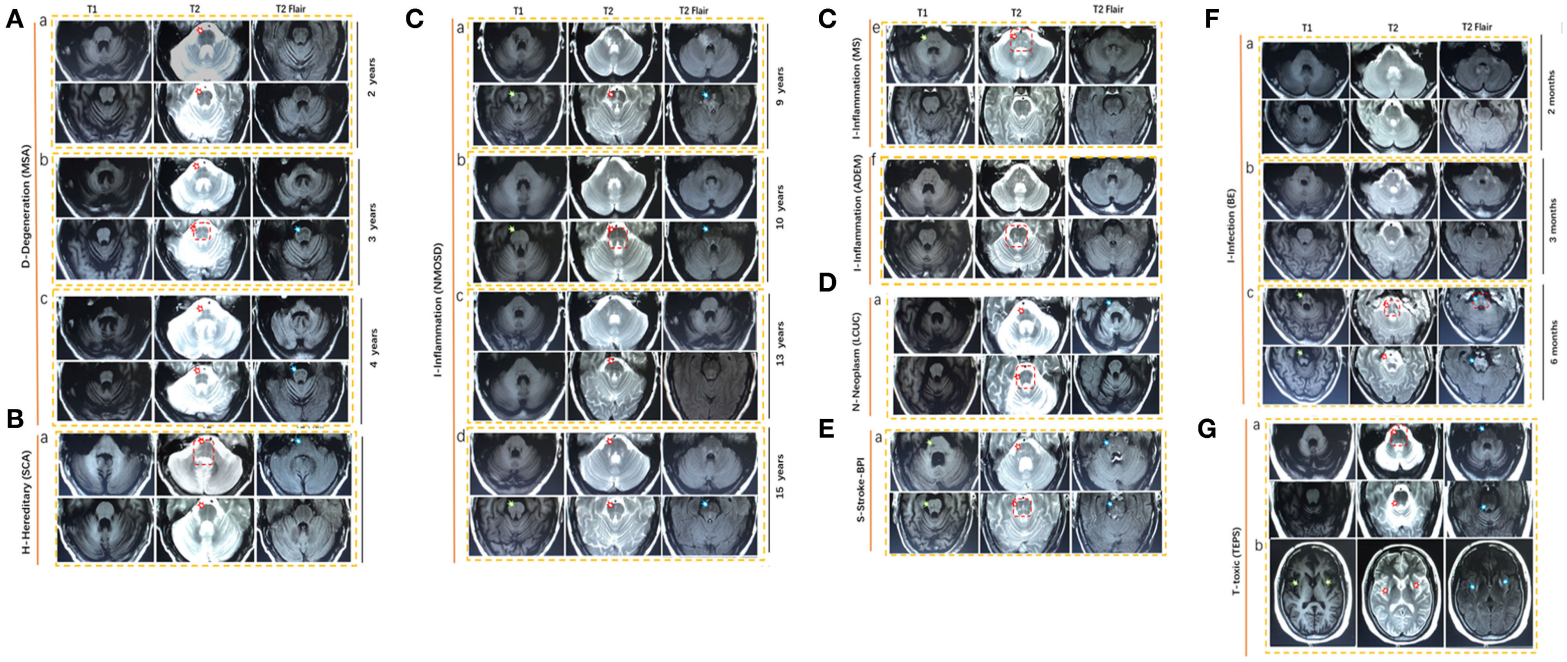
Representative magnetic resonance imaging (MRI) scans in nine HCBS-positive patients with **(A)** degenerative diseases (MSA), **(B)** hereditary disease (SCA), **(C)** inflammation, **(D)** neoplasm, **(E)** stroke, **(F)** infection, and **(G)** toxic. MSA, multiple system atrophy; NMOSD, neuromyelitis optica spectrum disorders; MS, multiple sclerosis; ADEM, acute disseminated encephalomyelitis; BE, brainstem encephalitis; LCUC, lung cancer with undefined cause; BPI, bilateral middle cerebral peduncle infarction; SCA, spinocerebellar ataxia; TEPS, toxic encephalopathy caused by phenytoin sodium.

The clinical heterogeneity is likely influenced by the underlying conditions. For example, MSA-C patients usually display severe cerebellar ataxia accompanied by a clear HCBs signal on T2-weighted images (Figure 5) as the disease progresses. The severity of clinical symptoms is consistent with the clarity of the HCBs. However, in some patients (cases N1 to N11) with infection and inflammation, cerebellar symptoms or signs were usually absent despite a clear HCBs in MR imaging. In MSA patients, a high signal of the HCBs was detected only on T2-weighted MRI; however, in some inflammatory diseases, a high signal could be found not only on T2 but also on T2 FLAIR-weighted MRI (cases N1, 3, and 8). In addition, in MSA cases, we noticed that no treatment was effective in attenuating MR HCBs signals, but in inflammatory diseases, immunotherapy can lead to the reduction or even disappearance of the HCBs signal (cases N1, N3, and N10). However, when the disease recurred, the HCBs signal reappeared or was enhanced (case N1). These findings strongly suggest that inflammatory demyelination of pontocerebellar fibers, but not merely gliosis, was responsible for the occurrence of the HCBs in inflammatory diseases (Figure 3).

Another published case ^44^ (case P39) and one newly screened patient in our center (case N40) were diagnosed with bilateral pons infarction and severe stenosis of the bilateral vertebral artery. In case N40, 1 month after the onset of infarction, the HCBs appeared in the pons; however, this patient had no cerebellar symptoms. The MR signal of the HCBs showed hypointensity on T1, hyperintensity on T2 and T2 Flair, and no signal on the DWI sequence. We think that the HCBs in this patient was likely due to Wallerian degeneration after ischemia rather than recurrence of stroke.

Our study has some inherent limitations. The hospital-based setting of our study may have resulted in a selection bias and hence likely underestimation of the general prevalence of HCBs. In the future study, we would like to carry out a prospective multicenter study to further expand the disease spectrums of HCBS and clarify the characteristics of HCBs in various diseases. The diagnosis of MSA in our cohort was based on clinical diagnostic criteria, with no postmortem confirmation. However, we have comprehensively studied a large clinical cohort together with reviewing published cases. Our study has certain strengths. We have completely and systemically reviewed previously published cases from PubMed searching and investigated a relatively large number of patients with the HCBs in our cohort. In this study, we firstly reported that HCBs was seen in a patient with NMOSD. According to the treatment response, we infer that HCBs in the NMOSD patient was caused by inflammatory demyelination, rather than by degenerative diseases such as MSA. Moreover, besides patients with NMOSD, we also found similar lesions in patients with other inflammatory demyelinating diseases, such as MS and ADEM. This finding implies that the pontocerebellar fiber as indicated by HCBs may be another predisposing site in inflammatory demyelinating diseases. However, to clarify the deduction, pathological results from autopsy are needed in the future.

## Conclusion

In summary, our findings suggest that the HCBs is more widely distributed than previously recognized. According to the clinical characteristics and neuroimaging features, there could be different neuro-pathogenesis involved in HCBs occurrence. There are still many unsolved confusions about the HCBs. For example, what are the gliosis components in the HCBs in MSA patients? Is the activation of microglia or astrocytes or the deposition of oligodendroglia cytoplasmic involved in the gliosis of cerebellopontine tract and occurrence of the HCBs? In the future, more autopsy and pathological research need to be done to obtain a better understanding of the HCBs.

## Data Availability Statement

The raw data supporting the conclusions of this article will be made available by the authors, without undue reservation.

## Ethics Statement

The studies involving human participants were reviewed and approved by the Ethics Committees of the Zhujiang Hospital of Southern Medical University, China. The patients/participants provided their written informed consent to participate in this study. Written informed consent was obtained from the individual(s) for the publication of any potentially identifiable images or data included in this article.

## Author Contributions

QW and SZ: Conceived and designed the study. SZ, HL, BD, and ZC: Performed the study. E-KT, JZ, ZH, YH, ZW, YL, MY, L-LC: Revised the paper for intellectual content. SZ, YL, L-LC, and BD: Data statistics and analysis. SZ, E-KT, and QW: Wrote the paper. All authors read and approved the final manuscript.

## Conflict of Interest

The authors declare that the research was conducted in the absence of any commercial or financial relationships that could be construed as a potential conflict of interest.

## Abbreviations

ADEM: acute disseminated encephalomyelitis
BC: breast cancer
BE: brainstem encephalitis
BPI: bilateral middle cerebral peduncle infarction
CJD: variant Creutzfeldt-Jakob disease
CNS: central nervous system
CSF: cerebrospinal fluid
CST: corticospinal tracts
CX: cerebrotendinous Xanthomatosis
EMRS: electronic medical record system
HCBS: ‘Hot cross bun’ sign
HIV-PML: HIV related progressive multifocal leukoencephalopathy
LC: lung cancer
LCUC: lung cancer with undefined cause
MS: multiple sclerosis
MSA: multiple system atrophy
MRI: Magnetic resonance imaging
NMOSD: neuromyelitis optica spectrum disorders
PD: Parkinson's disease
PSP: Progressive superanuclear palsy
RIS: radiologic information system
SCA: spinocerebellar ataxia
TEPS: Toxic encephalopathy caused by phenytoin sodium
UMSARS: Unified Multiple System Atrophy Rating Scale.
