# Supplementary material for: Various Diseases and Clinical Heterogeneity Are Associated With “Hot Cross Bun”
Source: Front Aging Neurosci. 2020 Nov 20;12:592212. doi: 10.3389/fnagi.2020.592212 (PMC7714952; doi:10.3389/fnagi.2020.592212)
Supplement: Supplementary file 1 [file Table_1.DOC]

| **Disease Spectrum**  **Table S1. Demographic information, clinical phenotypes and neuroimaging features of published and newly patients with HCBs** | **Case** | **Essential information** | | | **Clinical manifestation** | | **MRI information** | | | | | | | | **Treatment＆ Response** | | | **Source** |
| --- | --- | --- | --- | --- | --- | --- | --- | --- | --- | --- | --- | --- | --- | --- | --- | --- | --- | --- |
|  |  | **Age** | **G** | **DD** | **Main manifestation** | **Symptoms**  **“A”, “P”,“C“** | **Grade** | **Sequence** | | | **MCP- H** | **P-A** | **MCP-A** | **Cerebellar atrophy** | **Drug** | **Response** | **Follow-up** |  |
|  |  |  |  |  |  |  |  | **T1** | **T2** | **T2 Flair** |  |  |  |  |  |  |  |  |
| **M-metabolism** |  |  |  |  |  |  |  |  |  |  |  |  |  |  |  |  |  |  |
| 1. CTX | P1 | 25y | M | 17y | Cognitive decline, Cerebellar ataxia, Spastic quadriparesis, cataract and tendo-Achilles swelling (xanthoma) | C | 3 | N/A | **+** | N/A | N/A | No atrophy | N/A | Hyperintensities in the bilateral dentate nuclei | Chenodeoxycholic acid | N/A |  | Jain, R. S. India，2013[^1^](#_ENREF_1) |
| **I-inflammation** |  |  |  |  |  |  |  |  |  |  |  |  |  |  |  |  |  |  |
| 1. NMOSD | N1 | 55y | F | 18d | Weakness and numbness of the lower limbs | No | 4 | **+** | **+** | **+** | **+** | No atrophy | No atrophy | No atrophy | Methylprednisolone | Disappear | **+** |  |
|  | N2 | 38y | F | 1m | Numbness of waist | No | 3 | **-** | **+** | **-** | **-** | No atrophy | No atrophy | No atrophy | Methylprednisolone | N/A |  |  |
|  | N3 | 51y | M | 7d | Blurred vision | No | 3 | **-** | **+** | **+** | **-** | No atrophy | No atrophy | No atrophy | Methylprednisolone | Disappear | **+** |  |
|  | N4 | 46y | F | 5d | Weakness of lower limbs | No | 1 | **-** | **+** | **-** | **-** | No atrophy | No atrophy | No atrophy | Methylprednisolone | N/A |  |  |
|  | N5* | 56y | F | 2m | Weakness of limbs and blurred vision | No | 4 | **+** | **+** | **-** | **-** | No atrophy | No atrophy | No atrophy | Methylprednisolone | N/A |  |  |
| 1. MS | N6 | 49y | M | 9m | Bradyphasia, cognitive decline, unclear articulation | No | 1 | **+** | **+** | **-** | **+** | No atrophy | No atrophy | No atrophy | Methylprednisolone | N/A |  |  |
|  | N7 | 62y | F | 5m | Weakness of limbs and blurred vision | No | 1 | **-** | **+** | **-** | **-** | No atrophy | No atrophy | No atrophy | Methylprednisolone | N/A |  |  |
|  | N8 | 48y | F | 4m | Weakness of right limb | No | 1 | **-** | **+** | **+** | **-** | No atrophy | No atrophy | No atrophy | Methylprednisolone | N/A |  |  |
|  | N9 | 26y | M | 6m | Vision loss of left eye | No | 3 | **-** | **+** | **-** | **-** | No atrophy | No atrophy | No atrophy | Methylprednisolone | N/A |  |  |
| 1. ADEM | N10 | 54y | M | 3w | Fever, weakness of limbs | No | 1 | **+** | **+** | **-** | **-** | No atrophy | No atrophy | No atrophy | Methylprednisolone | Disappear | **+** |  |
|  | N11 | 40y | M | 2w | Fever, unconsciousness, weakness of limbs | No | 3 | **-** | **+** | **-** | **-** | No atrophy | No atrophy | No atrophy | Methylprednisolone | N/A |  |  |
| **D-degeneration** |  |  |  |  |  |  |  |  |  |  |  |  |  |  |  |  |  |  |
| 1. DLB | P2 | 60y | M | 5y | Dementia& Parkinsonism | P | 2 | N/A | **+** | N/A | **-** | Atrophy | Atrophy | Atrophy | N/A | N/A |  | Way, C. India,2019[^2^](#_ENREF_2) |
| 1. PSPV | P3 | 31y | F | 1.5y | Parkinsonism | P | 5 | N/A | **+** | N/A | **+** | Atrophy | Atrophy | Atrophy | ACTH | N/A |  | Muqit MM. UK,2001[^3^](#_ENREF_3) |
| 1. MSA | P4 | 60y | M | 2y | Cerebellar ataxia & Parkinsonism | C&P | N/A | N/A | N/A | N/A | N/A | N/A | N/A | N/A | N/A | N/A |  | Way, C. India，2019[^2^](#_ENREF_2) |
|  | P5 | 66y | M | 3y | Cerebellar ataxia & Parkinsonism | C&P | N/A | N/A | N/A | N/A | N/A | N/A | N/A | N/A | N/A | N/A |  | Way, C. India，2019[^2^](#_ENREF_2) |
|  | P6 | 69y | F | 8y | Cerebellar ataxia & Parkinsonism | C&P | N/A | N/A | N/A | N/A | N/A | N/A | N/A | N/A | N/A | N/A |  | Way, C. India，2019[^2^](#_ENREF_2) |
|  | P7 | 68y | F | 3y | Cerebellar ataxia & Parkinsonism | C&P | N/A | N/A | N/A | N/A | N/A | N/A | N/A | N/A | N/A | N/A |  | Way, C. India，2019[^2^](#_ENREF_2) |
|  | P8 | 65y | F | 4y | Cerebellar ataxia & Parkinsonism& Urinary incontinence | C&P&U | N/A | N/A | N/A | N/A | N/A | N/A | N/A | N/A | N/A | N/A |  | Way, C. India，2019[^2^](#_ENREF_2) |
|  | P9 | 68y | F | N/A | Cerebellar ataxia & Parkinsonism | C&P | N/A | N/A | N/A | N/A | N/A | N/A | N/A | N/A | N/A | N/A |  | Way, C. India，2019[^2^](#_ENREF_2) |
|  | P10 | 69y | M | 0.83y | Cerebellar ataxia | C | 1 | N/A | **+** | N/A | **+** | Atrophy | Atrophy | Atrophy | N/A | N/A |  | Lin, C. W. Taiwan,2016[^4^](#_ENREF_4) |
|  | P11 | 46y | M | N/A | N/A | N/A | 4 | N/A | **+** | N/A | **-** | Atrophy | Atrophy | Atrophy | N/A | N/A |  | Das, B. India,2016[^5^](#_ENREF_5) |
|  | P12 | 64y | M | 2y | Cerebellar ataxia | C | 3 | N/A | **+** | N/A | **-** | Atrophy | Atrophy | Atrophy | N/A | N/A |  | Das, B. India,2016[^5^](#_ENREF_5) |
|  | P13 | 60y | F | 3.5y | Parkinsonism | P | 4 | N/A | **+** | N/A | **-** | Atrophy | Atrophy | Atrophy | N/A | N/A |  | Srivastava, T. India,2005[^6^](#_ENREF_6) |
|  | P14 | 53y | M | N/A | Cerebellar ataxia | C | 4 | N/A | **+** | N/A | **-** | Atrophy | Atrophy | Atrophy | N/A | N/A |  | Srivastava, T. India,2005[^6^](#_ENREF_6) |
|  | P15 | 43y | M | N/A | Parkinsonism | P | 3 | N/A | **+** | N/A | **-** | Atrophy | Atrophy | Atrophy | N/A | N/A |  | Takao, M. Japan,2007[^7^](#_ENREF_7) |
|  | P16 | 59y | M | 2y | Cerebellar ataxia | C | 3 | N/A | **+** | N/A | **-** | Atrophy | Atrophy | Atrophy | N/A | N/A |  | Massano J Portugal,2008[^8^](#_ENREF_8) |
|  | P17 | 62y | F | 3y | Cerebellar ataxia & Parkinsonism& Urinary incontinence | C&P&U | 3 | N/A | **+** | N/A | **-** | Atrophy | Atrophy | Atrophy | N/A | N/A |  | Recio M. Spain,2012[^9^](#_ENREF_9) |
|  | P18 | 74y | F | 6y | Parkinsonism | P | 3 | N/A | **+** | N/A | **-** | Atrophy | Atrophy | Atrophy | N/A | N/A |  | Recio M. Spain,2012[^9^](#_ENREF_9) |
|  | P19 | 85y | M | 3y | Cerebellar ataxia & Parkinsonism | C&P | 3 | N/A | **+** | N/A | **-** | Atrophy | Atrophy | Atrophy | N/A | N/A |  | Recio M. Spain,2012[^9^](#_ENREF_9) |
|  | P20 | 60y | M | 3y | Cerebellar ataxia | C | 3 | N/A | **+** | N/A | **-** | Atrophy | Atrophy | Atrophy | N/A | N/A |  | Suresh CJ, India,2008[^10^](#_ENREF_10) |
|  | N12 | 61y | F | 5y | Cerebellar ataxia | C | 5 | **+** | **+** | **-** | **+** | Atrophy | Atrophy | Atrophy | N/A | N/A |  |  |
|  | N13 | 61y | F | 3y | Cerebellar ataxia | C | 2 | **-** | **+** | **-** | **-** | Atrophy | Atrophy | Atrophy | N/A | N/A |  |  |
|  | N14 | 63y | M | 3y | Cerebellar ataxia | C | 2 | **-** | **+** | **-** | **-** | Atrophy | Atrophy | Atrophy | N/A | N/A |  |  |
|  | N15 | 50y | F | 3y | Bradykinesia | P | 1 | **-** | **+** | **-** | **-** | Atrophy | Atrophy | Atrophy | N/A | N/A |  |  |
|  | N16 | 58y | M | 3y | Static tremor and unclear articulation | P | 1 | **-** | **+** | **-** | **-** | Atrophy | Atrophy | Atrophy | N/A | N/A |  |  |
|  | N17 | 41y | F | 2m | Rigidity of right lower extremity | P | 3 | **-** | **+** | **-** | **-** | Atrophy | Atrophy | Atrophy | N/A | N/A |  |  |
|  | N18 | 62y | F | 8m | Cerebellar ataxia | C | 4 | **-** | **+** | **-** | **+** | Atrophy | Atrophy | Atrophy | N/A | N/A |  |  |
|  | N19 | 21y | F | 1y | Cerebellar ataxia & unclear articulation | C | 1 | **-** | **+** | **-** | **-** | Atrophy | Atrophy | Atrophy | N/A | N/A |  |  |
|  | N20 | 57y | F | 9m | Cerebellar ataxia | C | 4 | **-** | **+** | **-** | **-** | Atrophy | Atrophy | Atrophy | N/A | N/A |  |  |
|  | N21 | 40y | M | 2y | Cerebellar ataxia & dizzy | C | 5 | **-** | **+** | **-** | **-** | Atrophy | Atrophy | Atrophy | N/A | N/A |  |  |
|  | N22 | 50y | F | 1y | Cerebellar ataxia | C | 4 | **-** | **+** | **-** | **-** | Atrophy | Atrophy | Atrophy | N/A | N/A |  |  |
|  | N23 | 55y | F | 3y | Bradykinesia & Rigidity | P | 3 | **-** | **+** | **-** | **-** | No atrophy | Atrophy | Atrophy | N/A | N/A |  |  |
|  | N24 | 64y | F | 1y | Cerebellar ataxia | C | 3 | **-** | **+** | **-** | **-** | No atrophy | Atrophy | Atrophy | N/A | N/A |  |  |
|  | N25 | 49y | M | 4m | Cerebellar ataxia & dizzy | C | 3 | **-** | **+** | **-** | **-** | Atrophy | Atrophy | Atrophy | N/A | N/A |  |  |
|  | N26 | 61y | F | 4y | Bradykinesia & Cerebellar ataxia | C&P | 1 | **-** | **+** | **-** | **-** | Atrophy | Atrophy | Atrophy | N/A | N/A |  |  |
|  | N27 | 52y | F | 0.5y | Cerebellar ataxia & dizzy | C | 1 | **-** | **+** | **-** | **-** | No atrophy | No atrophy | Atrophy | N/A | N/A |  |  |
|  | N28 | 65y | F | 4y | Bradykinesia | P | 3 | **-** | **+** | **-** | **-** | Atrophy | Atrophy | Atrophy | N/A | N/A |  |  |
|  | N29 | 70y | F | 1y | Cerebellar ataxia & unclear articulation | C | 4 | **-** | **+** | **-** | **-** | Atrophy | Atrophy | Atrophy | N/A | N/A |  |  |
|  | N30 | 59y | F | 5y | Cerebellar ataxia | C | 2 | **-** | **+** | **-** | **-** | Atrophy | Atrophy | Atrophy | N/A | N/A |  |  |
|  | N31 | 52y | F | 1y | Cerebellar ataxia & Bradykinesia & Rigidity | C&P | 3 | **-** | **+** | **-** | **-** | Atrophy | Atrophy | Atrophy | N/A | N/A |  |  |
|  | N32 | 55y | F | 3y | Bradykinesia & Rigidity | P | 2 | **-** | **+** | **-** | **-** | No atrophy | Atrophy | Atrophy | N/A | N/A |  |  |
|  | N33 | 56y | F | 5y | Cerebellar ataxia | C | 4 | **-** | **+** | **-** | **-** | Atrophy | Atrophy | Atrophy | N/A | N/A |  |  |
|  | N34 | 61y | F | 0.5y | Cerebellar ataxia | C | 1 | **-** | **+** | **-** | **-** | No atrophy | Atrophy | Atrophy | N/A | N/A |  |  |
| **N-neoplasm** |  |  |  |  |  |  |  |  |  |  |  |  |  |  |  |  |  |  |
| 1. PNSTT | P21 | 42y | M | N/A | Cerebellar ataxia | C | 4 | N/A | **+** | N/A | **-** | Atrophy | Atrophy | Atrophy | N/A | N/A |  | Ishikawa H, Japan 2016[^11^](#_ENREF_11) |
| 1. LMBC | P22 | 58y | F | N/A | No symptom | No | 3 | N/A | **+** | N/A | - | No atrophy | No atrophy | No atrophy | N/A | N/A |  | Pan, Z. China,2015[^12^](#_ENREF_12) |
| 1. LC | P23 | 38y | F | N/A | No symptom | No | 3 | N/A | **+** | **-** | **+** | Atrophy | Atrophy | Atrophy | N/A | N/A |  | Zhang, H. China,2013[^13^](#_ENREF_13) |
| 1. Neurosarcoid-osis | P24 | 48y | M | 0.3y | Cerebellar ataxia | C | 4 | N/A | **+** | N/A | **+** | Atrophy | Atrophy | Atrophy | N/A | N/A |  | Nagpal, K. India,2017[^14^](#_ENREF_14) |
| 1. LCUC | N35 | 79y | F | 2y | Routine review and no symptom | No | 4 | **-** | **+** | N/A | **+** | Atrophy | Atrophy | Atrophy | N/A | N/A |  |  |
| **I-infection** |  |  |  |  |  |  |  |  |  |  |  |  |  |  |  |  |  |  |
| 1. Encephalitis | P25 | 3y | M | N/A | N/A | N/A | N/A | N/A | **+** | N/A | **-** | No atrophy | No atrophy | No atrophy | Immunosuppressive | Disappear |  | Gan, Y. China，2018[^15^](#_ENREF_15) |
| 1. PML-HIV | P26 | 42y | M | 0.17y | Cerebellar ataxia | C | 4 | **-** | **+** | **+** | **+** | No atrophy | No atrophy | No atrophy | N/A | N/A |  | Jain RS. India,2014[^16^](#_ENREF_16) |
|  | P27 | 37y | F | 0.17y | Cerebellar ataxia | C | 4 | **-** | **+** | **+** | N/A | No atrophy | No atrophy | T2 hyperintensity  No atrophy | N/A | N/A |  | Padmanabhan,India,2013[^17^](#_ENREF_17) |
|  | P28 | 29y | F | 0.17y | Cerebellar ataxia | C | 3 | N/A | **+** | **+** | **+** | No atrophy | No atrophy | T2 hyperintensity  No atrophy | N/A | N/A |  | Yadav R.India,2011[^18^](#_ENREF_18) |
|  | P29 | 32y | M | 0.25y | Cerebellar ataxia | C | 3 | **+** | **+** | **+** | **+** | No atrophy | No atrophy | T2 hyperintensity  No atrophy | N/A | N/A |  | Yadav R.India,2011[^18^](#_ENREF_18) |
|  | P30 | 13y | F | 1.5y | Persistent vegetative state | No | 3 | N/A | N/A | **+** | **+** | Atrophy | Atrophy | Atrophy | N/A | N/A |  | Soares,J.P. Portugal, 2009[^19^](#_ENREF_19) |
| 1. BE | N36 | 30y | M | 8w | Coma and dyspnea | No | 3 | **+** | **+** | **+** | **-** | No atrophy | No atrophy | Atrophy | Methylprednisolone | N/A |  |  |
| **H-hereditary** |  |  |  |  |  |  |  |  |  |  |  |  |  |  |  |  |  |  |
| 1. SCA1 | P31 | 79y | M | 1y | Cerebellar ataxia & Dysarthria | C | 3 | N/A | **+** | N/A | **-** | Atrophy | Atrophy | Atrophy | N/A | N/A |  | Wang, Y. Japan,2016[^20^](#_ENREF_20) |
|  | P32 | 37y | M | 5y | Cerebellar ataxia & Dementia | C | 3 | N/A | **+** | N/A | **-** | Atrophy | Atrophy | Atrophy | N/A | N/A |  | Namekawa, M Japan,2015[^21^](#_ENREF_21) |
|  | P33 | 35y | M | N/A | Cerebellar ataxia & Dementia | C | 2 | N/A | **+** | N/A | **-** | Atrophy | Atrophy | Atrophy | N/A | N/A |  | Namekawa, M Japan,2015[^21^](#_ENREF_21) |
| 1. SCA3 | P34 | 59y | M | 9y | Cerebellar ataxia & Urinary incontinency | C& A | 3 | N/A | **+** | **+** | **+** | Atrophy | Atrophy | Atrophy | N/A | N/A |  | Pedroso, J. L Brazil,2013[^22^](#_ENREF_22) |
| 1. SCA2 | P35 | 20y | M | 6y | Cerebellar ataxia | C | 2 | N/A | **+** | N/A | **+** | No atrophy | No atrophy | No atrophy | N/A | N/A |  | Gooneratne, I. K. India, 2013[^23^](#_ENREF_23) |
| 1. SCA(?)^e^ | P36 | 40y | F | 2y | Cerebellar ataxia& Parkinsonism | C& P | 3 | N/A | **+** | N/A | **+** | Atrophy | Atrophy | Atrophy | N/A | N/A |  | Way, C. India,2019[^2^](#_ENREF_2) |
|  | P37 | 79y | M | 33y | Cerebellar ataxia& Parkinsonism | C& P | 1 | N/A | **+** | N/A | **+** | Atrophy | Atrophy | Atrophy | N/A | N/A |  | Way, C. India,2019[^2^](#_ENREF_2) |
|  | P38 | 68y | M | 36y | Cerebellar ataxia& Parkinsonism | C& P | 2 | N/A | **+** | N/A | **+** | Atrophy | Atrophy | Atrophy | N/A | N/A |  | Way, C. India,2019[^2^](#_ENREF_2) |
|  | N37 | 39y | M | 6y | Cerebellar ataxia&, dysarthria | C | 3 | **+** | **+** | **+** | **-** | Atrophy | Atrophy | Atrophy | N/A | N/A |  |  |
|  | N38 | 55y | F | 5y | Cerebellar ataxia & numbness of lower limbs | C | 3 | **+** | **+** | **-** | **-** | No atrophy | Atrophy | Atrophy | N/A | N/A |  |  |
| **T-****Toxication** |  |  |  |  |  |  |  |  |  |  |  |  |  |  |  |  |  |  |
| 1. TEPS | N39 | 24y | F | 20y | Epilepsy, limb weakness, Cerebellar ataxia | N/A | 3 | **-** | **+** | **-** | **-** | No atrophy | Atrophy | Atrophy | N/A | N/A |  |  |
| **S-stroke** |  |  |  |  |  |  |  |  |  |  |  |  |  |  |  |  |  |  |
| 1. BPI | P39 | 71y | F | 1y | Action tremor | No | 3 | N/A | **+** | N/A | **-** | No atrophy | No atrophy | No atrophy | Antiplatelet | N/A |  | Roh, SY. South Korea, 2013[^24^](#_ENREF_24) |
|  | N40 | 61y | M | 4m | Limb weakness and numbness | No | 3 | N/A | **+** | N/A | **-** | No atrophy | No atrophy | No atrophy | Antiplatelet | N/A |  |  |

Abbreviation: ADEM = Acute disseminated encephalomyelitis; BE = Brainstem encephalitis ; BPI = Bilateral pontine infarction ; C= Cerebellar ataxia; CTX, Cerebrotendinous Xanthomatosis; F = Female; G= gender; DLB, dementia with Lewy body; DD= disease duration; HCBs = Hot cross bun sign; LC, lung cancer; LMBC; Leptomeningeal metastases of breast cancer; LCUC, lung cancer with undefined cause; M = male; MS = Multiple sclerosis; MCP-H: middle cerebellar peduncle hyperintensity; P-A: pons atrophy; MCP-A: middle cerebellar peduncle atrophy; MSA = Multiple system atrophy; N/A = not available; NMOSD = Neuromyelitis Optica spectrum disorders; PNSTT, Paraneoplastic neurological syndrome due to burned-out testicular tumor; PSPV, Parkinsonism secondary to presumed vasculitis; PML-HIV = Progressive multifocal leukoencephalopathy with HIV seropositivity; SCA = Spinocerebellar ataxia; TEPS, Toxic encephalopathy caused by phenytoin sodium; UD = Urinary dysfunction; d = day; m = month; y = year.

**References**

1. Jain RS, Sannegowda RB, Agrawal A, Hemrajani D, Jain R, Mathur T. 'Hot cross bun' sign in a case of cerebrotendinous xanthomatosis: a rare neuroimaging observation. BMJ case reports 2013;2013.

2. Way C, Pettersson D, Hiller A. The 'Hot Cross Bun' Sign Is Not Always Multiple System Atrophy: Etiologies of 11 Cases. Journal of movement disorders 2019;12:27-30.

3. Muqit MM, Mort D, Miskiel KA, Shakir RA. "Hot cross bun" sign in a patient with parkinsonism secondary to presumed vasculitis. Journal of neurology, neurosurgery, and psychiatry 2001;71:565-566.

4. Lin CW, Tseng CY, Lo CP, Tu MC. A Case of Multiple System Atrophy with Preexisting Alzheimer's Disease and Predating The Hot Cross Bun Sign. Acta neurologica Taiwanica 2016;25(4):152-159.

5. Das B, Patil A, Goyal MK, Modi M, Lal V, Ahuja C. 'Hot cross bun' sign. QJM : monthly journal of the Association of Physicians 2016;109:203-204.

6. Srivastava T, Singh S, Goyal V, Shukla G, Behari M. "Hot cross bun" sign in two patients with multiple system atrophy-cerebellar. Neurology 2005;64:128.

7. Takao M, Kadowaki T, Tomita Y, Yoshida Y, Mihara B. 'Hot-cross bun sign' of multiple system atrophy. Internal medicine 2007;46:1883.

8. Massano J, Costa F, Nadais G. Teaching neuroImage: MRI in multiple system atrophy: "hot cross bun" sign and hyperintense rim bordering the putamina. Neurology 2008;71:e38.

9. Recio Bermejo M, Navarro Munoz S, Espejo Martinez B, Mangas Aveleira C, Garcia Ruiz R. ["Hot-cross bun sign" in multiple system atrophy: a presentation of 3 cases]. Neurologia 2012;27:314-315.

10. Suresh Chandran CJ, Godge YR, Oak PJ, Ravat SH. Hot cross bun sign. Neurology India 2008;56:218.

11. Ishikawa H, Kawada N, Taniguchi A, et al. Paraneoplastic neurological syndrome due to burned-out testicular tumor showing hot cross-bun sign. Acta neurologica Scandinavica 2016;133:398-402.

12. Pan Z, Yang G, Yuan T, et al. 'Hot cross bun' sign with leptomeningeal metastases of breast cancer: a case report and review of the literature. World journal of surgical oncology 2015;13:43.

13. Zhang H, Tian Y, Jin T, Zhang H, Sun L. The "hot cross bun" sign in leptomeningeal carcinomatosis. The Canadian journal of neurological sciences Le journal canadien des sciences neurologiques 2013;40:597-598.

14. Nagpal K, Agarwal P. "Hot-cross bun" and "inverse trident sign' in neurosarcoidosis: An important finding. Neurology India 2017;65:175-176.

15. Gan Y, Liang H, Li X, et al. The hot cross bun sign in a patient with encephalitis. Brain & development 2018;40:503-506.

16. Jain RS, Nagpal K, Tejwani S. 'Hot-cross bun' and 'inverse trident sign' in progressive multifocal leukoencephalopathy with HIV seropositivity. Neurology India 2014;62:341-342.

17. Padmanabhan S, Cherian A, Iype T, Mathew M, Smitha S. Hot cross bun sign in HIV-related progressive multifocal leukoencephalopathy. Annals of Indian Academy of Neurology 2013;16:672-673.

18. Yadav R, Ramdas M, Karthik N, et al. "Hot cross bun" sign in HIV-related progressive multifocal leukoencephalopathy. Neurology India 2011;59:293-294.

19. Soares-Fernandes JP, Ribeiro M, Machado A. "Hot cross bun" sign in variant Creutzfeldt-Jakob disease. AJNR American journal of neuroradiology 2009;30:E37.

20. Wang Y, Koh K, Takaki R, Shindo K, Takiyama Y. Hot cross bun sign in a late-onset SCA1 patient. Neurological sciences : official journal of the Italian Neurological Society and of the Italian Society of Clinical Neurophysiology 2016;37:1873-1874.

21. Namekawa M, Honda J, Shimazaki H. "Hot cross bun" sign associated with SCA1. Internal medicine 2015;54:859-860.

22. Pedroso JL, Rivero RL, Barsottini OG. "Hot cross bun" sign resembling multiple system atrophy in a patient with Machado-Joseph disease. Arquivos de neuro-psiquiatria 2013;71:824.

23. Gooneratne IK, Caldera MC, Perera SP, Gamage R. Hot cross bun sign in a patient with cerebellar ataxia. Annals of Indian Academy of Neurology 2013;16:406.

24. Roh SY, Jang HS, Kim YH. Hot cross bun sign following bilateral pontine infarction: a case report. Journal of movement disorders 2013;6:37-39.

**1). Supplementary References (78)**

1. Carre G, Dietemann JL, Gebus O, et al. Brain MRI of multiple system atrophy of cerebellar type: a prospective study with implications for diagnosis criteria. Journal of neurology 2020.
2. Way C, Pettersson D, Hiller A. The 'Hot Cross Bun' Sign Is Not Always Multiple System Atrophy: Etiologies of 11 Cases. Journal of movement disorders 2019;12:27-30.
3. Tsuda M, Asano S, Kato Y, Murai K, Miyazaki M. Differential diagnosis of multiple system atrophy with predominant parkinsonism and Parkinson's disease using neural networks. Journal of the neurological sciences 2019;401:19-26.
4. Sugiyama A, Sekiguchi Y, Beppu M, Ishige T, Matsushita K, Kuwabara S. Diagnostic Challenges Posed by Preceding Peripheral Neuropathy in Very Late-onset Spinocerebellar Ataxia Type 3. Internal medicine 2019;58:119-122.
5. Portet M, Filyridou M, Howlett DC. Hot cross bun sign. Journal of neurology 2019;266:2573-2574.
6. Ozaki K, Ansai A, Nobuhara K, et al. Prevalence and clinicoradiological features of spinocerebellar ataxia type 34 in a Japanese ataxia cohort. Parkinsonism & related disorders 2019;65:238-242.
7. Meira AT, Arruda WO, Ono SE, et al. Neuroradiological Findings in the Spinocerebellar Ataxias. Tremor and other hyperkinetic movements 2019;9.
8. Li Q, Sun R, Song Q, et al. [Effects of different fluid replenishment methods on internal environment, body thermal regulation response and severe heatstroke of 5-km armed cross-country training soldiers]. Zhonghua wei zhong bing ji jiu yi xue 2019;31:1028-1032.
9. Kim M, Ahn JH, Cho Y, Kim JS, Youn J, Cho JW. Differential value of brain magnetic resonance imaging in multiple system atrophy cerebellar phenotype and spinocerebellar ataxias. Scientific reports 2019;9:17329.
10. Chelban V, Bocchetta M, Hassanein S, Haridy NA, Houlden H, Rohrer JD. An update on advances in magnetic resonance imaging of multiple system atrophy. Journal of neurology 2019;266:1036-1045.
11. Nishimori M, Murata Y, Iwasa H, et al. Comparison of MRI and (123)I-FP-CIT SPECT for the evaluation of MSA-P clinical severity. Biomedical reports 2018;8:523-528.
12. Li X, Zhou C, Cui L, et al. A case of a novel CACNA1G mutation from a Chinese family with SCA42: A case report and literature review. Medicine 2018;97:e12148.
13. Krishnan M, Balamurugan N, Mayan V, Nidhin PD. Hot Cross Bun Sign - Multisystem Atrophy (Cerebellar Type). The Journal of the Association of Physicians of India 2018;66:87.
14. Higashi M, Ozaki K, Hattori T, et al. A diagnostic decision tree for adult cerebellar ataxia based on pontine magnetic resonance imaging. Journal of the neurological sciences 2018;387:187-195.
15. Gan Y, Liang H, Li X, et al. The hot cross bun sign in a patient with encephalitis. Brain & development 2018;40:503-506.
16. Yamasaki R, Yamaguchi H, Matsushita T, Fujii T, Hiwatashi A, Kira JI. Early strong intrathecal inflammation in cerebellar type multiple system atrophy by cerebrospinal fluid cytokine/chemokine profiles: a case control study. Journal of neuroinflammation 2017;14:89.
17. Pradhan S, Tandon R. Relevance of non-specific MRI features in multiple system atrophy. Clinical neurology and neurosurgery 2017;159:29-33.
18. Nagpal K, Agarwal P. "Hot-cross bun" and "inverse trident sign' in neurosarcoidosis: An important finding. Neurology India 2017;65:175-176.
19. Kuwabara M, Hisatome I, Roncal-Jimenez CA, et al. Increased Serum Sodium and Serum Osmolarity Are Independent Risk Factors for Developing Chronic Kidney Disease; 5 Year Cohort Study. PloS one 2017;12:e0169137.
20. Cicilet S, Furruqh F, Biswas A, Philip B. Hot cross bun and bright middle cerebellar peduncle signs in cerebellar type multiple system atrophy. BMJ case reports 2017;2017.
21. Budhram A, Pelikan JB, Kremenchutzky M, Sharma M. The 'across the pons' sign: A possible novel radiographic finding in natalizumab-associated progressive multifocal leukoencephalopathy. Journal of the neurological sciences 2017;375:304-306.
22. Wesseling C, Aragon A, Gonzalez M, et al. Heat stress, hydration and uric acid: a cross-sectional study in workers of three occupations in a hotspot of Mesoamerican nephropathy in Nicaragua. BMJ open 2016;6:e011034.
23. Wesseling C, Aragon A, Gonzalez M, et al. Kidney function in sugarcane cutters in Nicaragua--A longitudinal study of workers at risk of Mesoamerican nephropathy. Environmental research 2016;147:125-132.
24. Wang Y, Koh K, Takaki R, Shindo K, Takiyama Y. Hot cross bun sign in a late-onset SCA1 patient. Neurological sciences : official journal of the Italian Neurological Society and of the Italian Society of Clinical Neurophysiology 2016;37:1873-1874.
25. Lin CW, Tseng CY, Lo CP, Tu MC. A Case of Multiple System Atrophy with Preexisting Alzheimer's Disease and Predating The Hot Cross Bun Sign. Acta neurologica Taiwanica 2016;25(4):152-159.
26. Jain RS, Kumar S, Tejwani S. Medullary Hot-Cross Bun Sign in Multiple System Atrophy-Cerebellar. Journal of medical imaging and radiation sciences 2016;47:113-115.
27. Ishikawa H, Kawada N, Taniguchi A, et al. Paraneoplastic neurological syndrome due to burned-out testicular tumor showing hot cross-bun sign. Acta neurologica Scandinavica 2016;133:398-402.
28. Das B, Patil A, Goyal MK, Modi M, Lal V, Ahuja C. 'Hot cross bun' sign. QJM : monthly journal of the Association of Physicians 2016;109:203-204.
29. Alsemari A, Al-Hindi HN. Large-scale mitochondrial DNA deletion underlying familial multiple system atrophy of the cerebellar subtype. Clinical case reports 2016;4:111-117.
30. Saigoh K, Mitsui J, Hirano M, et al. The first Japanese familial case of spinocerebellar ataxia 23 with a novel mutation in the PDYN gene. Parkinsonism & related disorders 2015;21:332-334.
31. Pan Z, Yang G, Yuan T, et al. 'Hot cross bun' sign with leptomeningeal metastases of breast cancer: a case report and review of the literature. World journal of surgical oncology 2015;13:43.
32. Ozaki K, Doi H, Mitsui J, et al. A Novel Mutation in ELOVL4 Leading to Spinocerebellar Ataxia (SCA) With the Hot Cross Bun Sign but Lacking Erythrokeratodermia: A Broadened Spectrum of SCA34. JAMA neurology 2015;72:797-805.
33. Namekawa M, Honda J, Shimazaki H. "Hot cross bun" sign associated with SCA1. Internal medicine 2015;54:859-860.
34. Deguchi K, Ikeda K, Kume K, et al. Significance of the hot-cross bun sign on T2*-weighted MRI for the diagnosis of multiple system atrophy. Journal of neurology 2015;262:1433-1439.
35. Rohani M. Hot cross bun sign in a case with multisystem atrophy. Iranian journal of neurology 2014;13:110-111.
36. Peeraully T. Multiple system atrophy. Seminars in neurology 2014;34:174-181.
37. Jain RS, Sannegowda RB, Jain R, Prakash S. Reverse 'hot cross bun', 'Mercedes-Benz', 'face of the giant panda and her cub' signs with pontine infarcts: a radiological pandora. BMJ case reports 2014;2014.
38. Jain RS, Nagpal K, Tejwani S. 'Hot-cross bun' and 'inverse trident sign' in progressive multifocal leukoencephalopathy with HIV seropositivity. Neurology India 2014;62:341-342
39. Goldman JS, Quinzii C, Dunning-Broadbent J, et al. Multiple system atrophy and amyotrophic lateral sclerosis in a family with hexanucleotide repeat expansions in C9orf72. JAMA neurology 2014;71:771-774.
40. Baeza Trinidad R, Serrano Ponz M. [Multiple system atrophy: hot cross bun and Santiaguino sign]. Revista clinica espanola 2014;214:e9.
41. Zhang H, Tian Y, Jin T, Zhang H, Sun L. The "hot cross bun" sign in leptomeningeal carcinomatosis. The Canadian journal of neurological sciences Le journal canadien des sciences neurologiques 2013;40:597-598.
42. Watanabe H, Senda J, Ito M, et al. [Cutting-edge MRI techniques for studying neurological diseases focusing on spinocerebellar degeneration]. Rinsho shinkeigaku = Clinical neurology 2013;53:1087-1090.
43. Roh SY, Jang HS, Kim YH. Hot cross bun sign following bilateral pontine infarction: a case report. Journal of movement disorders 2013;6:37-39.
44. Pedroso JL, Rivero RL, Barsottini OG. "Hot cross bun" sign resembling multiple system atrophy in a patient with Machado-Joseph disease. Arquivos de neuro-psiquiatria 2013;71:824.
45. Padmanabhan S, Cherian A, Iype T, Mathew M, Smitha S. Hot cross bun sign in HIV-related progressive multifocal leukoencephalopathy. Annals of Indian Academy of Neurology 2013;16:672-673.
46. Jain RS, Sannegowda RB, Agrawal A, Hemrajani D, Jain R, Mathur T. 'Hot cross bun' sign in a case of cerebrotendinous xanthomatosis: a rare neuroimaging observation. BMJ case reports 2013;2013.
47. Gooneratne IK, Caldera MC, Perera SP, Gamage R. Hot cross bun sign in a patient with cerebellar ataxia. Annals of Indian Academy of Neurology 2013;16:406.
48. Recio Bermejo M, Navarro Munoz S, Espejo Martinez B, Mangas Aveleira C, Garcia Ruiz R. ["Hot-cross bun sign" in multiple system atrophy: a presentation of 3 cases]. Neurologia 2012;27:314-315.
49. Massey LA, Micallef C, Paviour DC, et al. Conventional magnetic resonance imaging in confirmed progressive supranuclear palsy and multiple system atrophy. Movement disorders : official journal of the Movement Disorder Society 2012;27:1754-1762.
50. Kasahara S, Miki Y, Kanagaki M, et al. "Hot cross bun" sign in multiple system atrophy with predominant cerebellar ataxia: a comparison between proton density-weighted imaging and T2-weighted imaging. European journal of radiology 2012;81:2848-2852.
51. Hohler AD, Singh VJ. Probable hereditary multiple system atrophy-autonomic (MSA-A) in a family in the United States. Journal of clinical neuroscience : official journal of the Neurosurgical Society of Australasia 2012;19:479-480.
52. Brooks DJ. Parkinson's disease: diagnosis. Parkinsonism & related disorders 2012;18 Suppl 1:S31-33.
53. Yadav R, Ramdas M, Karthik N, et al. "Hot cross bun" sign in HIV-related progressive multifocal leukoencephalopathy. Neurology India 2011;59:293-294.
54. Watanabe H, Yoshida M, Naganawa S, Sobue G. [The diagnosis of neurodegenerative disorders based on clinical and pathological findings using an MRI approach]. Rinsho shinkeigaku = Clinical neurology 2011;51:863-864.
55. Loh KB, Rahmat K, Lim SY, Ramli N. A Hot Cross Bun sign from diffusion tensor imaging and tractography perspective. Neurology India 2011;59:266-269.
56. Fujimori J, Tatewaki Y, Shimizu H, Kimura I, Hisanaga K. [Degeneration of ponto-cerebellar tract visualized by diffusion tensor imaging in multiple system atrophy]. Rinsho shinkeigaku = Clinical neurology 2011;51:271-274.
57. Baronica KB, Ivkic G, Ozretic D, Milicevic G. Differential diagnostic relevance of high resolution magnetic resonance in patients with possible multiple system atrophy (MSA) - A case report. Collegium antropologicum 2011;35 Suppl 1:287-292.
58. Segwe A. Hot cross bun anyone? European neurology 2010;64:360.
59. Haugbol S, Rasmussen BK, Reith J. [Picture of the month: hot cross bun sign]. Ugeskrift for laeger 2010;172:1465.
60. de Mello RA, Ferreira D, Dias da Costa JM, Rosas MJ, Quinaz JM. Multiple-system atrophy with cerebellar predominance presenting as respiratory insufficiency and vocal cords paralysis. Case reports in medicine 2010;2010.
61. Damon-Perriere N, Tison F, Meissner WG. [Multiple system atrophy]. Psychologie & neuropsychiatrie du vieillissement 2010;8:179-191.
62. Soares-Fernandes JP, Ribeiro M, Machado A. "Hot cross bun" sign in variant Creutzfeldt-Jakob disease. AJNR American journal of neuroradiology 2009;30:E37.
63. Marrannes J, Mulleners E. Hot cross bun sign in a patient with SCA-2. JBR-BTR : organe de la Societe royale belge de radiologie 2009;92:263.
64. Lee YC, Liu CS, Wu HM, Wang PS, Chang MH, Soong BW. The 'hot cross bun' sign in the patients with spinocerebellar ataxia. European journal of neurology 2009;16:513-516.
65. Kimura N, Kumamoto T, Masuda T, et al. Evaluation of regional cerebral blood flow in cerebellar variant of multiple system atrophy using FineSRT. Clinical neurology and neurosurgery 2009;111:829-834.
66. Gulati A, Virmani V, Singh P, Khandelwal N. The hot cross bun sign. Neurology India 2009;57:104-105.
67. Suresh Chandran CJ, Godge YR, Oak PJ, Ravat SH. Hot cross bun sign. Neurology India 2008;56:218.
68. Massano J, Costa F, Nadais G. Teaching neuroImage: MRI in multiple system atrophy: "hot cross bun" sign and hyperintense rim bordering the putamina. Neurology 2008;71:e38.
69. Juyal R, Chaurasia R, Malhotra HS, Shukla R. "Hot cross bun" sign. The Journal of the Association of Physicians of India 2008;56:986.
70. Takao M, Kadowaki T, Tomita Y, Yoshida Y, Mihara B. 'Hot-cross bun sign' of multiple system atrophy. Internal medicine 2007;46:1883.
71. Shrivastava A. The hot cross bun sign. Radiology 2007;245:606-607.
72. Ito M, Watanabe H, Kawai Y, et al. Usefulness of combined fractional anisotropy and apparent diffusion coefficient values for detection of involvement in multiple system atrophy. Journal of neurology, neurosurgery, and psychiatry 2007;78:722-728.
73. Bhidayasiri R, Perlman S. Medical image. "Hot cross bun" sign. The New Zealand medical journal 2007;120:U2563.
74. Srivastava T, Singh S, Goyal V, Shukla G, Behari M. "Hot cross bun" sign in two patients with multiple system atrophy-cerebellar. Neurology 2005;64:128.
75. Watanabe H, Fukatsu H, Katsuno M, et al. Multiple regional 1H-MR spectroscopy in multiple system atrophy: NAA/Cr reduction in pontine base as a valuable diagnostic marker. Journal of neurology, neurosurgery, and psychiatry 2004;75:103-109.
76. Watanabe H, Saito Y, Terao S, et al. Progression and prognosis in multiple system atrophy: an analysis of 230 Japanese patients. Brain : a journal of neurology 2002;125:1070-1083.
77. Muqit MM, Mort D, Miskiel KA, Shakir RA. "Hot cross bun" sign in a patient with parkinsonism secondary to presumed vasculitis. Journal of neurology, neurosurgery, and psychiatry 2001;71:565-566.
78. Youvan D, Watanabe M, Holmquist R. Morphology of extremely heat-resistant spores from Bacillus sp. ATCC 27380 by scanning and transmission electron microscopy. Life sciences and space research 1977;15:65-72.

**2). Relative References (24)**

1. Jain RS, Sannegowda RB, Agrawal A, Hemrajani D, Jain R, Mathur T. 'Hot cross bun' sign in a case of cerebrotendinous xanthomatosis: a rare neuroimaging observation. BMJ case reports 2013;2013.

2. Way C, Pettersson D, Hiller A. The 'Hot Cross Bun' Sign Is Not Always Multiple System Atrophy: Etiologies of 11 Cases. Journal of movement disorders 2019;12:27-30.

3. Muqit MM, Mort D, Miskiel KA, Shakir RA. "Hot cross bun" sign in a patient with parkinsonism secondary to presumed vasculitis. Journal of neurology, neurosurgery, and psychiatry 2001;71:565-566.

4. Lin CW, Tseng CY, Lo CP, Tu MC. A Case of Multiple System Atrophy with Preexisting Alzheimer's Disease and Predating The Hot Cross Bun Sign. Acta neurologica Taiwanica 2016;25(4):152-159.

5. Das B, Patil A, Goyal MK, Modi M, Lal V, Ahuja C. 'Hot cross bun' sign. QJM : monthly journal of the Association of Physicians 2016;109:203-204.

6. Srivastava T, Singh S, Goyal V, Shukla G, Behari M. "Hot cross bun" sign in two patients with multiple system atrophy-cerebellar. Neurology 2005;64:128.

7. Takao M, Kadowaki T, Tomita Y, Yoshida Y, Mihara B. 'Hot-cross bun sign' of multiple system atrophy. Internal medicine 2007;46:1883.

8. Massano J, Costa F, Nadais G. Teaching neuroImage: MRI in multiple system atrophy: "hot cross bun" sign and hyperintense rim bordering the putamina. Neurology 2008;71:e38.

9. Recio Bermejo M, Navarro Munoz S, Espejo Martinez B, Mangas Aveleira C, Garcia Ruiz R. ["Hot-cross bun sign" in multiple system atrophy: a presentation of 3 cases]. Neurologia 2012;27:314-315.

10. Suresh Chandran CJ, Godge YR, Oak PJ, Ravat SH. Hot cross bun sign. Neurology India 2008;56:218.

11. Ishikawa H, Kawada N, Taniguchi A, et al. Paraneoplastic neurological syndrome due to burned-out testicular tumor showing hot cross-bun sign. Acta neurologica Scandinavica 2016;133:398-402.

12. Pan Z, Yang G, Yuan T, et al. 'Hot cross bun' sign with leptomeningeal metastases of breast cancer: a case report and review of the literature. World journal of surgical oncology 2015;13:43.

13. Zhang H, Tian Y, Jin T, Zhang H, Sun L. The "hot cross bun" sign in leptomeningeal carcinomatosis. The Canadian journal of neurological sciences Le journal canadien des sciences neurologiques 2013;40:597-598.

14. Nagpal K, Agarwal P. "Hot-cross bun" and "inverse trident sign' in neurosarcoidosis: An important finding. Neurology India 2017;65:175-176.

15. Gan Y, Liang H, Li X, et al. The hot cross bun sign in a patient with encephalitis. Brain & development 2018;40:503-506.

16. Jain RS, Nagpal K, Tejwani S. 'Hot-cross bun' and 'inverse trident sign' in progressive multifocal leukoencephalopathy with HIV seropositivity. Neurology India 2014;62:341-342.

17. Padmanabhan S, Cherian A, Iype T, Mathew M, Smitha S. Hot cross bun sign in HIV-related progressive multifocal leukoencephalopathy. Annals of Indian Academy of Neurology 2013;16:672-673.

18. Yadav R, Ramdas M, Karthik N, et al. "Hot cross bun" sign in HIV-related progressive multifocal leukoencephalopathy. Neurology India 2011;59:293-294.

19. Soares-Fernandes JP, Ribeiro M, Machado A. "Hot cross bun" sign in variant Creutzfeldt-Jakob disease. AJNR American journal of neuroradiology 2009;30:E37.

20. Wang Y, Koh K, Takaki R, Shindo K, Takiyama Y. Hot cross bun sign in a late-onset SCA1 patient. Neurological sciences : official journal of the Italian Neurological Society and of the Italian Society of Clinical Neurophysiology 2016;37:1873-1874.

21. Namekawa M, Honda J, Shimazaki H. "Hot cross bun" sign associated with SCA1. Internal medicine 2015;54:859-860.

22. Pedroso JL, Rivero RL, Barsottini OG. "Hot cross bun" sign resembling multiple system atrophy in a patient with Machado-Joseph disease. Arquivos de neuro-psiquiatria 2013;71:824.

23. Gooneratne IK, Caldera MC, Perera SP, Gamage R. Hot cross bun sign in a patient with cerebellar ataxia. Annals of Indian Academy of Neurology 2013;16:406.

24. Roh SY, Jang HS, Kim YH. Hot cross bun sign following bilateral pontine infarction: a case report. Journal of movement disorders 2013;6:37-39.
